# Racial Residential Segregation and Race Differences in Ideal Cardiovascular Health among Young Men

**DOI:** 10.3390/ijerph18157755

**Published:** 2021-07-22

**Authors:** Samuel L. K. Baxter, Richard Chung, Leah Frerichs, Roland J. Thorpe, Asheley C. Skinner, Morris Weinberger

**Affiliations:** 1 Department of Public Health Sciences, Clemson University, Clemson, SC 29634, USA; 2 Department of Pediatrics, Duke University School of Medicine, Duke University, Durham, NC 27710, USA; richard.chung@duke.edu; 3 Department of Health Policy and Management, Gillings School of Global Public Health, University of North Carolina at Chapel Hill, Chapel Hill, SC 27599, USA; leahf@email.unc.edu (L.F.); mweinber@email.unc.edu (M.W.); 4 Hopkins Center for Health Disparities Solutions, Program for Research on Men’s Health, Johns Hopkins Bloomberg School of Public Health, Baltimore, MD 21205, USA; rthorpe@jhu.edu; 5 Department of Population Health Sciences, Duke University School of Medicine, Durham, NC 27710, USA; asheley.skinner@duke.edu

**Keywords:** cardiovascular health, residential segregation, health disparities, young adulthood

## Abstract

Background: Race disparities in cardiovascular disease (CVD) related morbidity and mortality are evident among men. While previous studies show health in young adulthood and racial residential segregation (RRS) are important factors for CVD risk, these factors have not been widely studied in male populations. We sought to examine race differences in ideal cardiovascular health (CVH) among young men (ages 24–34) and whether RRS influenced this association. Methods: We used cross-sectional data from young men who participated in Wave IV (2008) of the National Longitudinal Survey of Adolescent to Adult Health (*N* = 5080). The dichotomous outcome, achieving ideal CVH, was defined as having ≥4 of the American Heart Association’s Life’s Simple 7 targets. Race (Black/White) and RRS (proportion of White residents in census tract) were the independent variables. Descriptive and multivariate analyses were conducted. Results: Young Black men had lower odds of achieving ideal CVH (OR = 0.67, 95% CI = 0.49, 0.92) than young White men. However, RRS did not have a significant effect on race differences in ideal CVH until the proportion of White residents was ≥55%. Conclusions: Among young Black and White men, RRS is an important factor to consider when seeking to understand CVH and reduce future cardiovascular risk.

## 1. Introduction

Cardiovascular disease (CVD) is the leading cause of death for all adults in the US, with the death rate being higher in men than women [1,2]. Men’s excess burden can also be seen in higher rates of CVD-related morbidity, risk-taking, and poor health care engagement compared to women [3]. Notably, a stark racial disparity in CVD mortality is observed between Black and White men 25–34 years old (21.1 vs. 9.8 deaths/100,000 population) [2].

Given the population-level CVD burden in the US and recognition of the importance of early life years in disease trajectories, the American Heart Association (AHA) expanded CVD prevention efforts to focus on maintaining health. To this end, the AHA outlined 7 targets, termed Life’s Simple 7 (LS7), with the goal of improving the cardiovascular health (CVH) of Americans by 20% [4]. The LS7 targets of ideal CVH are healthy diet, moderate to vigorous physical activity, no smoking, normal body mass index (BMI), and normal blood pressure, total cholesterol, and blood glucose levels without taking prescription medication. CVH has a strong inverse association with CVD morbidity [5,6]. Fewer than 20% of adults have ideal CVH, and there are differences by race and biological sex. White adults are nearly twice as likely to meet the criteria for ideal CVH than Black adults (19.4% vs. 10.6%), and women are more likely to achieve ideal CVH than men (22% vs. 15%) [7]. These differences in CVH reflect disparities in genetic, social, and environmental CVD risk factors.

One explanation for racial disparities in the prevalence of CVD risk factors is racial residential segregation (RRS) [8,9], defined as the degree to which two or more racial groups live in separate environments [10]. Notable scholars on racism and health consider RRS an upstream determinant that creates and sustains racial disparities in health [11,12,13]. Unfortunately, much of the literature on racial disparities in health fails to acknowledge that most Americans live in segregated environments [14]. Minorities living in segregated, majority–minority environments endure structural barriers to educational and employment opportunities, increased exposure to social stressors, and differential access to resources that promote health [10,15]. A recent literature review suggests that RRS has deleterious effects on CVD risk for Black adults [16]. The interplay of RRS, race, and health has gained increased attention in men’s health disparities literature. A community-based study found no racial disparity in CVD risk factors (obesity, physical inactivity, hypertension, smoking, diabetes) when White and Black men lived in the same environment [17,18].

Most evidence supporting segregation effects on race differences in CVD focuses on middle-aged adults and excludes young adults. This is concerning because young adulthood is an important period for establishing health practices that shape health in later adult years [19,20]. Prior studies demonstrate that being healthy in young adulthood is associated with lower CVD risk in middle-age [21,22]. However, the impact of residential context on young adults’ CVD risk is understudied. Furthermore, the CVH trajectories of today’s young adults may be affected by growing up during the childhood obesity epidemic, having many modifiable CVD risk factors, and being disproportionately uninsured [23]. Though limited, recent data show that 18% of young adults have ideal CVH, with Blacks and men less likely to have ideal CVH than Whites and women, respectively [24]. To date, Black-White disparities in CVH among young adults have been attributed to differences in educational attainment, insurance status, and neighborhood population density [5,24,25]. These factors are unequally distributed across race and residential patterns. To our knowledge, no study has examined the relationship between RRS and CVH disparities in young adult men.

Given these research gaps, the goal of this study was to understand Black-White differences in ideal CVH among young men and the contribution of RRS to these differences. Our interest in the relationship between RRS, race, and ideal CVH was guided by Williams’ (1997) seminal framework that explicates the additive and interactive processes in which race differences in health are generated by fundamental causes and observed as surface causes in research. Societal forces (e.g., culture, geography, racism, economic structures, and political factors) and biology are positioned as fundamental causes of variations in health. As fundamental causes, changes in societal forces bring about changes in the conditions that affect health at individual, community, and population levels. As Williams and Collins (2001) argue, past and present RRS is a societal force that maintains the conditions through which health disparities persist. RRS—created by federal policies that have functioned in American society for centuries through discriminatory housing market practices and policies—is a manifestation of structural racism [11]. RRS spatially concentrates Black Americans in residential areas with limited access to the resources and opportunities necessary to promote and protect health [9].

Additionally, this framework posits race as a complex, multidimensional construct that bears the historical consequences of multiple large-scale societal structures and processes that support racism at multiple levels [12]. Race, among other social status categories (e.g., socioeconomic status, age, gender), is inscribed with political and societal powers and privileges that link the fundamental causes to racial differences in health outcomes through surface causes. Surface causes include a range of factors (e.g., neighborhood context, risk behaviors, stressful life events, medical care, psychosocial factors) that correlate with each other to result in differential health outcomes by race. Like social status categories, surface causes are shaped by larger societal structures and processes. Although surface causes are the usual intervention targets to address race differences in health, they are likely insufficient without intervening on the fundamental causes. For example, a surface intervention to address race disparities in smoking may focus on stress management and smoking cessation. However, intervening on smoking disparities through fundamental causes may include passing policies that limit the presence of tobacco product advertisements (e.g., billboards, store window advertisements, events) in minority communities. Thus, developing effective strategies that produce long-term population health impacts requires attention to both the surface and fundamental causes of race differences in health. Examining RRS may be an important step to understanding variations in ideal CVH by race in young men.

We hypothesize that among young men: (1) Whites will be more likely to have ideal CVH than Blacks; (2) those living in neighborhoods with a higher proportion of White residents will be more likely to have ideal CVH; (3) living in neighborhoods with a higher proportion of White residents will be associated with Black-White differences in ideal CVH.

## 2. Materials and Methods

### 2.1. Data Source and Sample

Data came from Wave IV (2008) of the National Longitudinal Study of Adolescent to Adult Health (Add Health), a nationally-representative cohort study of adolescents (grades 7–12) who were followed into adulthood. Add Health used a multistage, stratified, clustered sampling design where schools were systematically sampled to reflect the diversity of US adolescents with respect to census region, school type and size, urbanicity, and proportion of White students. Adolescents were sampled from 80 high schools and 52 middle schools to complete home interviews in 1994–1995 (Wave I) and 1996 (Wave II) [26]. Follow-up interviews were conducted in 2001–2002 (Wave III) and 2008–2009 (Wave IV). The most recent wave of data collection was completed in 2016–2018 (Wave V). Add Health oversampled for Black respondents with highly educated parents and paired respondent interview data with contextual data on aspects of their residential environment at each wave.

Our study included Black and White men who did not simultaneously identify with any other racial or ethnicity categories, had a valid sampling weight, and did not have missing values on the outcome variable or residential location (*n* = 5080). Wave IV included CVD-related biomarkers not measured in previous waves.

### 2.2. Dependent Variable

The dependent variable was ideal CVH, based on the AHA’s LS7 targets. First, we categorized respondents as having ideal, intermediate, or poor CVH for each LS7 target based on the definitions and thresholds provided in Supplementary Materials (Table S1). Next, we created and summed binary ideal CVH indicators for each LS7 target. We then created a binary ideal CVH variable representing whether or not respondents achieved ideal CVH for at least four of the LS7 targets. Respondents did not meet the criteria for ideal CVH if they reported a diagnosis of diabetes, hypertension, high cholesterol, or CVD. We chose this approach over a continuous or categorical (ideal, intermediate, and poor) CVH measure because it: (1) best aligned with the AHA’s desire to keep populations at the lowest risk of developing CVD by maintaining recommended levels of LS7 targets [4,27] and (2) was consistent with current literature operationalizing the AHA’s construct of ideal CVH [5,22,23,24]. Add Health trained staff to measure height, weight, and blood pressure, obtained blood glucose and total cholesterol from blood spots, and asked respondents about their diet, physical activity, and smoking practices. Additional information on data collection procedures is available at https://www.cpc.unc.edu/projects/addhealth/documentation/guides (last accessed on 1 June 2021).

### 2.3. Independent Variables

The primary independent variables of interest were race and RRS. Race was categorized as White (referent group) or Black. RRS was defined as the ratio of White residents in the neighborhood to total census tract population where the participant resided at during Wave IV. Consistent with previous work, neighborhoods were defined using census tract boundaries [8,28].

### 2.4. Covariates

We included two sets of factors informed by existing literature and the study’s framework: social status and surface causes (neighborhood context, risk behavior, stressful life event, medical care, and psychosocial factors). Social status included age at time of interview and two dimensions of socioeconomic status: educational attainment (1 = less than high school; 2 = high school graduate; 3 = some college; 4 = college degree or more) and income-to-needs ratio (ratio of self-reported household income to poverty threshold for that year and household size based on the US Census). Given the diversity of neighborhood contexts in the US, we included census tract-level population density and urbanicity measured using rural-urban commuting area (RUCA) codes. RUCA codes classify US census tracts based on population density, level of urbanization, and daily commuting in relation to other census tracts. This classification can also be applied to zip codes. RUCA code categories were arranged as: 1 = Metropolitan neighborhood, 2 = Micropolitan neighborhood, and 3 = Small town/rural neighborhood. For risk behavior, we used a binary indicator of any binge drinking (consuming ≥5 drinks in a row in the past year). Stressful life events included self-report of ever being arrested, underemployment (working fewer than 10 h a week), and financial strain (6 questions assessed if respondents were unable to pay for phone service, food, utility bills, had a utility service turned off, full amount of rent or mortgage, or were evicted from their residence during the past year). Medical care included self-reports of being insured, having a routine health checkup in the past two years, and not obtaining medical care in the past year when needed. Finally, psychosocial factors included the 5-item Center for Epidemiological Studies Depression Scale (range 0–15) and Cohen’s 4-item perceived stress scale (range 0–16) [29,30].

### 2.5. Statistical Analysis

We did not impute missing values for ideal CVH or spatial geocodes. For covariates, we used multiple imputation chained equations for missing values. This allowed continuous and categorical variables to be imputed with their own specified distribution, rather than assuming one common distribution. Less than 7% of sample had missing information: 6% for the income-to-needs ratio and fewer than 1% for self-reported binge drinking, routine checkup, insurance status, unmet healthcare need, unemployment, financial strain, arrest experience, perceived stress, and depressive symptomology. To account for Add Health’s complex survey design and ensure representativeness, survey weights were applied when specifying statistical models.

We first calculated weighted descriptive statistics of the sample. In our logistic regression analysis of ideal CVH, we began with a baseline model that only included race. We then added RRS. Next, we adjusted for neighborhood context, social status, risk behavior, stressful life events, medical care, and psychosocial factors. Since population density was highly skewed, we used natural log transformation. Finally, we included an interaction term between race and RRS to assess whether the effect of RRS on ideal CVH differed by race. All analyses were conducted using STATA version 15 (StataCorp. 15, College Station, TX, USA) [31].

## 3. Results

Table 1 presents weighted descriptive statistics of the sample (*n* = 5080) by race. The average age of male respondents was 28.4 years (range 24–34 years), 21% of whom were Black. Approximately 27% of young men had ideal CVH, (≥4 LS7 targets at ideal levels). More White men had ideal CVH than Black men (28.0 versus 20.9, *p* = 0.014). On average, White men lived in neighborhoods with a higher proportion of White residents than Black men (81.4 ± 0.8 versus 47.7 ± 2.1, *p* = 0.000). Most men resided in metropolitan areas. White men were more likely to be college educated, have a higher income-to-needs ratio average, report binge drinking, and be insured. Black men were more likely to report receipt of a routine health checkup, financial strain, an arrest experience, and greater average scores for stress and depressive symptoms.

Table 2 presents analyses on the odds of having ideal CVH to test proposed hypotheses on race differences in ideal CVH and the role of RRS among young men.

**Hypothesis** **1.** *White men will be more likely to have ideal CVH than Black men.*

As hypothesized, at baseline, Black men had lower odds of ideal CVH (OR = 0.67, 95% CI = 0.49, 0.92) compared to White men (Table 2, Model 1). When adjusting for covariates (Table 2, Model 3), race remained significant in the model (OR = 0.70, 95% CI = 0.50, 0.97).

**Hypothesis** **2.** *Men who live in neighborhoods with a higher proportion of White residents will be more likely to have ideal CVH.*

When adding RRS to the unadjusted model (Table 2, Model 2), it was not a significant predictor of ideal CVH (OR = 0.99, 95% CI = 0.99, 1.00). When adjusting for covariates (Table 2, Model 3), the percentage of White residents in neighborhood was not significant in the model (OR = 1.00, 95% CI = 0.99, 1.00).

**Hypothesis** **3.** *Living in neighborhoods with a higher proportion of White residents will be associated with greater Black-White differences in ideal CVH among young men.*

When we added the interaction term between race and percentage of White residents in the neighborhood, race was no longer a significant predictor (Model 4, Table 2). This interaction term was not statistically significant (*p* = 0.098), indicating that a Black-White difference in ideal CVH was not observed at every RRS threshold.

**Figure 1:**
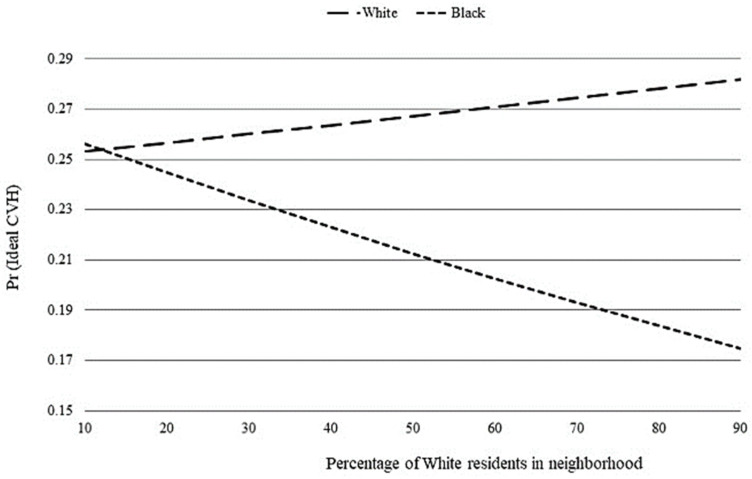
Figure graphically depicts race differences in ideal CVH along thresholds of racial residential segregation. Graph is based on Model 4 of Table 2. (1) Predicted probabilities of having ideal cardiovascular health (CVH) by percentage of White residents in neighborhood.

**Figure 2:**
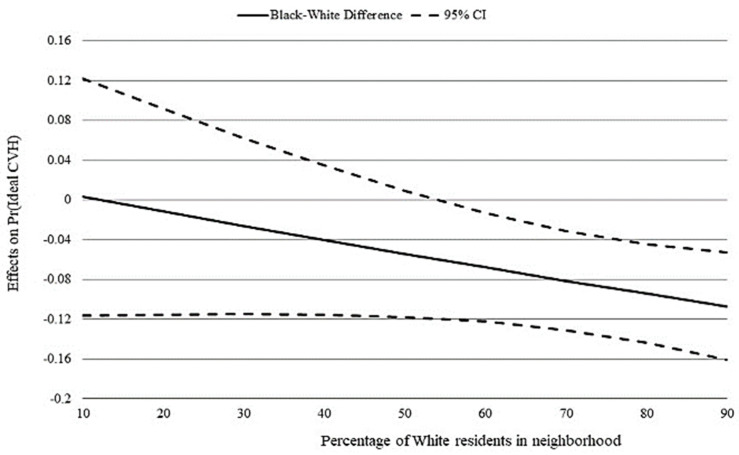
Figure graphically depicts race differences in ideal CVH along thresholds of racial residential segregation. Graph is based on Model 4 of Table 2. (2) Race differences in the marginal effect of percentage of White residents in neighborhood on ideal cardiovascular health (CVH). Dashed lines give 95% confidence interval (CI).

We next explored whether race differences in ideal CVH existed along RRS thresholds. As the percentage of White residents increased, the probability of having ideal CVH increased for White men but decreased for Black men (Figure 1). Specifically, the probability of having ideal CVH was statistically significant and lower for Black men than their White counterparts when the proportion of White residents was ≥55% (Figure 2). In contrast, there was no race difference when both groups lived where fewer than 55% of neighborhood residents were White.

## 4. Discussion

Increasing our understanding of race differences in CVH among young men is an important step towards reducing CVD disparities in later adult years. In a nationally-representative sample, we investigated if there was a Black-White difference in ideal CVH among young men (ages 24–34) and whether RRS contributed to any observed difference. We found significant race differences in both the proportion of young men with ideal CVH and the percentage of White residents in neighborhood. Young Black men had 0.3 lower odds of achieving ideal CVH than White men, and on average, young Black and White men lived in neighborhoods with markedly different racial compositions. Further, we observed that the probability of having ideal CVH increased for White, but decreased for Black, men as the proportion of White residents increased. However, RRS did not have a significant effect on race differences in ideal CVH until the proportion of White residents was ≥55%. Taken together, these findings demonstrate that a race disparity in CVH exists among young men and highlights RRS as an important determinant.

Ours is the first study to examine the role of RRS in race differences among young men. Our findings are consistent with research suggesting CVD disparities among men may begin in young adulthood [7] Further, this work fits within a growing body of evidence linking Black men’s higher CVD morbidity and mortality in middle-age to greater CVD risk as young adults [32]. Similar to most Americans, most of our sample lived in segregated environments. Ideal CVH declined among young Black men but increased among young White men as the proportion of White residents increased (Figure 1). This pattern of race differences is important because CVH declines have been associated with increased CVD risk [6]. Prior research in community-based studies demonstrated that higher proportions of Black residents was associated with increased CVD risk for middle-aged Black adults [16,33]. Our finding that young Black men are more likely to maintain ideal CVH when residing in majority–minority neighborhoods suggests that life stage may be an important factor to consider when examining the extent that RRS influences race disparities in CVH and CVD risk. Additionally, we found that Black men were less likely to have ideal CVH than White men only when both groups lived in neighborhoods with ≥55% White residents (Figure 2). In other words, a Black-White disparity existed when both groups lived in majority-White neighborhoods, but not when both groups lived in majority–minority neighborhoods. This finding supports prior research suggesting that health disparities dissipate when Black and White men live together in racially integrated neighborhoods under similar economic, social, and environmental conditions [14,17,18]. While it has been suggested that moving residents to better quality neighborhoods likely containing a higher proportion of White residents may improve cardiovascular outcomes for Black adults [16], our finding suggests this strategy may not improve the CVH of young Black men.

Of note, sample characteristics highlighted limited access to educational opportunities and excess exposure to stressors as mechanisms by which RRS upholds race disparities in this population [9,24]. Socioeconomic measures (i.e., education and income-to-needs ratio) favored White men while stressful life events (i.e., financial strain and arrest experience) and psychosocial factors (i.e., perceived stress and depressive symptoms) disfavored Black men. Components that disfavored young Black men align with prior research on factors that influence accelerated health declines among Black Americans [9,13,32]. We observed that more young Black men reported health care utilization (i.e., routine health checkup in the past two years) than young White men. This finding conflicts with prior research linking RRS to challenges regarding medical care access [10,16]. Because literature on RRS and health in young adults is limited, interaction effects were not explored in this paper but warrant further consideration in research exploring the link between racism, place, and various health outcomes among young men.

- Strengths and Limitations

Significant strengths of our study include a focus on CVH, a positive cardiovascular outcome, and RRS, a defining feature of racism in America. We acknowledge several limitations in our analysis. First, we defined ideal CVH as having at least four LS7 indicators, rather than five that other authors have used [24,25,34]. We selected four because detailed dietary data were not available to construct the LS7 target for diet [7]. Second, measuring RRS as racial composition in census tract, though widely used, has been criticized for not reflecting the relative distribution of racial groups within larger geographic areas (e.g., metropolitan statistical area or county) or spatial interaction patterns between racial groups [28]. Add Health does not include geographic identifiers that would allow us to calculate formal segregation measures and other neighborhood measures (e.g., area deprivation and social vulnerability indices) associated with health. Third, neighborhood context (population density and urbanicity) did not include features of the built and social environment, which may mediate the relationships explored in this study. Additionally, Add Health’s multistage sampling design did not include neighborhood-level indicators that would support multilevel analyses focused on neighborhood contexts. Lastly, our cross-sectional approach does not allow causal claims about observed associations.

Future studies on CVH outcomes should apply and compare formal and proxy RRS measures. We also recommend that future studies examine built, social, and economic features of segregated environments to better understand men’s CVH and associated race differences. There is a need for research that employs a multilevel framework to better understand the effects of RRS and CVH at the neighborhood and individual level [15]. Moreover, longitudinal and life course research approaches that examine the relationship between place, race, and CVH can offer insights into the causal mechanisms of CVH decline among men.

## 5. Conclusions

Our findings suggest young men may not be as healthy as they appear and RRS plays a significant role in young men’s CVH race disparities. Our findings emphasize a growing need to support structural interventions and policies to eliminate disparities in health and life chances by advancing racial equity where people live, work, and play across the life course. Bailey and colleagues [13] offer place-based, multisector, equity-oriented initiatives focused on eliminating structural barriers and critical analyses of racism in public health and medical education as important structural interventions and policies. Still, public health advocates are challenged to align equitable health protections with political interests outside the health sector. As a recent example, the Surgeon General’s Community Health and Economic Prosperity Report articulated that investing in community health is essential to business resilience and a thriving economy. Health care systems are well-positioned to deliver preventive services that can improve young men’s CVH. However, there is a need to increase men’s health care engagement and clinicians’ capacity to understand and translate knowledge of how residential context influences health into actionable strategies and equitable practices. Intentional changes in health care practices and policies are warranted to support young men’s CVH maintenance. When clinicians offer preventive health recommendations to young men, they should also inquire how the social determinants of health may make it easy or difficult to be healthy and follow recommendations. Advocates for men’s health equity call for gender mainstreaming in health care policies [3,35]. Gender mainstreaming considers the gendered perspectives of sex categories in policy processes and impacts. It acknowledges that gender-neutral policies fail men and women, do not use between-group gender comparisons to understand within-group gender health disparities, and support sex-disaggregated data analyses and reports to drive equitable healthy system changes [35]. In concert with these changes, modifying the social and built environments of segregated neighborhoods in ways that enable health, without causing displacement, may offset the emergence of race disparities in CVD risk development as young men age.

## Figures and Tables

**Table 1:** Weighted descriptive statistics of adult male participants by race, Add Health (Wave IV).

	Total (*n* = 5080)	White (*n* = 4001)	Black (*n* = 1079)	
Variables	% or Mean (SD)	% or Mean (SD)	% or Mean (SD)	*p*
Ideal cardiovascular health	26.9	28.0	20.9	0.014 ***
Percent White in neighborhood	76.1 (±1.306)	81.4 (±0.833)	47.7 (±2.154)	<0.001 ***
Neighborhood context				
Population density (persons/square km.)	1692 (±159)	1587 (±146)	2259 (±432)	0.123
Urbanicity				0.173
Metropolitan area	84.0	85.1	78.3	
Micropolitan area	10.2	9.5	14.0	
Small town/Rural area	5.8	5.4	8.7	
Social status				
Age	28.4 (±0.124)	28.4 (±0.131)	28.7 (±0.227)	0.116
Education				0.007 ***
Less than high school	10.1	9.4	13.9	
High school diploma	20.9	20.0	26.1	
Some college	43.0	43.0	42.7	
College degree or more	26.0	27.6	17.3	
Income-to-needs	4.5 (±0.093)	4.7 (±0.097)	3.5 (±0.138)	<0.001 ***
Risk behavior				
Binge drinking	79.6	81.0	71.6	<0.001 ***
Stressful life event				
Financial strain	22.8	21.1	32.0	<0.001 ***
Arrest experience	42.9	41.5	50.6	0.006 ***
Underemployed	29.5	28.6	33.9	0.077
Medical care				
Insurance status	73.1	74.5	65.2	<0.001 ***
Routine checkup	63.8	61.8	75.0	<0.001 ***
Unmet healthcare need	25.8	25.5	27.5	0.398
Psychosocial factors				
Perceived stress	4.5 (±0.064)	4.4 (±0.063)	5.0 (±0.168)	0.001 ***
Depressive symptoms	2.3 (±0.051)	2.2 (±0.047)	2.9 (±0.151)	<0.001 ***

*** *p* < 0.001. Analyses applied sampling weights and strata variables to account for sampling design.

**Table 2:** Logistic regression of odds of having ideal CVH ^a^ among 5080 adult male participants, Add Health (Wave IV).

	Model 1 ^b^	Model 2 ^b^	Model 3 ^c^	Model 4 ^c^
Variable	OR (95% CI)	OR (95% CI)	OR (95% CI)	OR (95% CI)
Black ^d^	0.67 (0.497, 0.924)	0.58 (0.415, 0.820)	0.70 (0.502, 0.970)	1.10 (0.525, 2.314)
Percent White in neighborhood ^e^		0.99 (0.991, 1.001)	1.00 (0.995, 1.004)	1.00 (0.995, 1.008)
Black × percent White in neighborhood ^f^				0.99 (0.982, 1.002)
Constant	0.39 (0.353, 0.428)	0.55 (0.370, 0.821)	0.21 (0.037, 1.137)	0.17 (0.028, 1.005)

Note. Ideal CVH = ideal cardiovascular health; OR = odds ratio; CI = confidence interval. ^a^ Ideal CVH is defined as 4 or more Life’s Simple 7 targets categorized as ideal; ^b^ Model does not adjust for covariates; ^c^ Model adjusts for neighborhood population density, neighborhood urbanicity (rural-urban commuting area code), age, educational attainment, income-to-needs ratio, binge drinking, financial strain, arrest experience, underemployment, insurance status, routine health checkup, unmet healthcare need, perceived stress, and depressive symptoms; ^d^ White men are the referent group; ^e^ Racial residential segregation proxy measure; ^f^ Mean percentage of White residents in neighborhood among White men is referent group; All estimates account for complex sampling design by applying appropriate sampling weights and strata variables.

## Data Availability

This study paired study participant’s data with contextual data files that were made available by a contractual restricted-use data agreement. To be eligible to enter into this contract, researchers must have an IRB-approved security plan for handling and storing sensitive data and sign a data-use contract agreeing to keep the data confidential. Data files were not downloaded and only accessed on a secure server.

## Supplementary Materials

The following is available online at https://www.mdpi.com/article/10.3390/ijerph18157755/s1, Table S1: Cardiovascular health: Ideal, intermediate, and poor thresholds of the Life’s Simple 7 targets.

## References

[1] Centers for Disease Control and Prevention, National Center for Health Statistics Underlying Cause of Death 1999–2018 on CDC WONDER Online Database, Released 2020. Data Are from the Multiple Cause of Death Files, 1999–2018, as Compiled from Data Provided by the 57 Vital Statistics Jurisdictions through the Vital Statistics Cooperative Program. http://wonder.cdc.gov/ucd-icd10.html.

[2] Heron M. (2019). Deaths: Leading Causes for 2017.

[3] Baker P., Shand T. (2017). Men’s health: Time for a new approach to policy and practice?. J. Glob. Health.

[4] Lloyd-Jones D.M., Hong Y., Labarthe D., Mozaffarian D., Appel L.J., Van Horn L., Greenlund K., Daniels S., Nichol G., Tomaselli G.F. (2010). Defining and setting national goals for cardiovascular health promotion and disease reduction: The American heart association’s strategic impact goal through 2020 and beyond. Circulation.

[5] McClurkin M.A., Yingling L.R., Ayers C., Cooper-McCann R., Suresh V., Nothwehr A., Barrington D.S., Powell-Wiley T.M. (2015). Health Insurance Status as a Barrier to Ideal Cardiovascular Health for U.S. Adults: Data from the National Health and Nutrition Examination Survey (NHANES). PLoS ONE.

[6] Yang Q., Cogswell M.E., Flanders W.D., Hong Y., Zhang Z., Loustalot F., Gillespie C., Merritt R., Hu F.B. (2012). Trends in Cardiovascular Health Metrics and Associations With All-Cause and CVD Mortality Among US Adults. JAMA.

[7] Benjamin E.J., Muntner P., Alonso A., Bittencourt M.S., Callaway C.W., Carson A.P., Chamberlain A.M., Chang A.R., Cheng S., Das S.R. (2019). Heart disease and stroke statistics—2019 update: A report from the American heart association. Circulation.

[8] Mujahid M.S., Moore L.V., Petito L.C., Kershaw K.N., Watson K., Roux A.V.D. (2017). Neighborhoods and racial/ethnic differences in ideal cardiovascular health (the Multi-Ethnic Study of Atherosclerosis). Health Place.

[9] Thorpe R.J., Fesahazion R.G., Parker L., Wilder T., Rooks R.N., Bowie J.V., Bell C.N., Szanton S.L., LaVeist T.A. (2016). Accelerated Health Declines among African Americans in the USA. J. Urban Health.

[10] Williams D.R., Collins C. (2001). Racial residential segregation: A fundamental cause of racial disparities in health. Public Health Rep..

[11] Riley A.R. (2017). Neighborhood Disadvantage, Residential Segregation, and Beyond—Lessons for Studying Structural Racism and Health. J. Racial Ethn. Health Disparities.

[12] Williams D.R. (1997). Race and health: Basic questions, emerging directions. Ann. Epidemiol..

[13] Bailey Z., Krieger N., Agénor M., Graves J., Linos N., Bassett M.T. (2017). Structural racism and health inequities in the USA: Evidence and interventions. Lancet.

[14] LaVeist T., Pollack K., Thorpe R., Fesahazion R., Gaskin D. (2011). Place, Not Race: Disparities Dissipate In Southwest Baltimore When Blacks And Whites Live Under Similar Conditions. Health Aff..

[15] Kershaw K.N., Osypuk T., Do D.P., de Chavez P.J., Roux A.V.D. (2015). Neighborhood-Level Racial/Ethnic Residential Segregation and Incident Cardiovascular Disease. Circulation.

[16] Kershaw K.N., Albrecht S.S. (2015). Racial/ethnic residential segregation and cardiovascular disease risk. Curr. Cardiovasc. Risk Rep..

[17] Thorpe R.J., Kelley E., Bowie J.V., Griffith D.M., Bruce M., LaVeist T. (2014). Explaining Racial Disparities in Obesity Among Men: Does Place Matter?. Am. J. Men Health.

[18] Thorpe R.J., Kennedy-Hendricks A., Griffith D., Bruce M.A., Coa K., Bell C.N., Young J., Bowie J.V., LaVeist T.A. (2015). Race, Social and Environmental Conditions, and Health Behaviors in Men. Fam. Community Health.

[19] Harris K.M., Gordon-Larsen P., Chantala K., Udry J.R. (2006). Longitudinal Trends in Race/Ethnic Disparities in Leading Health Indicators From Adolescence to Young Adulthood. Arch. Pediatr. Adolesc. Med..

[20] Stroud C., Walker L.R., Davis M., Irwin C.E. (2015). Investing in the Health and Well-Being of Young Adults. J. Adolesc. Health.

[21] Liu K., Daviglus M.L., Loria C.M., Colangelo L.A., Spring B., Moller A.C., Lloyd-Jones D. (2012). Healthy Lifestyle through Young Adulthood and the Presence of Low Cardiovascular Disease Risk Profile in Middle Age. Circulation.

[22] Unger E., Diez-Roux A.V., Lloyd-Jones D., Mujahid M.S., Nettleton J.A., Bertoni A., Badon S.E., Ning H., Allen N.B. (2014). Association of Neighborhood Characteristics with Cardiovascular Health in the Multi-Ethnic Study of Atherosclerosis. Circ. Cardiovasc. Qual. Outcomes.

[23] Lawrence E., Hummer R.A., Harris K.M. (2017). The Cardiovascular Health of Young Adults: Disparities along the Urban-Rural Continuum. Ann. Am. Acad. Polit. Soc. Sci..

[24] Lawrence E.M., Hummer R.A., Domingue B.W., Harris K.M. (2018). Wide educational disparities in young adult cardiovascular health. SSM Popul. Health.

[25] Lawrence E.M. (2017). Why Do College Graduates Behave More Healthfully Than Those Who Are Less Educated?. J. Health Soc. Behav..

[26] Harris K.M., Udry J.R. (2008). National Longitudinal Study of Adolescent to Adult Health (Add Health), 1994–2008 [Public Use]. ICPSR Data Holdings.

[27] Steinberger J., Daniels S.R., Hagberg N., Isasi C.R., Kelly A.S., Lloyd-Jones D., Pate R.R., Pratt C., Shay C.M., Towbin J.A. (2016). Cardiovascular Health Promotion in Children: Challenges and Opportunities for 2020 and Beyond: A Scientific Statement From the American Heart Association. Circulation.

[28] White K., Borrell L.N. (2011). Racial/ethnic residential segregation: Framing the context of health risk and health disparities. Health Place.

[29] Cohen S., Kamarck T., Mermelstein R. (1983). A global measure of perceived stress. J. Health Soc. Behav..

[30] Perreira K.M., Deeb-Sossa N., Harris K.M., Bollen K. (2005). What Are We Measuring? An Evaluation of the CES-D Across Race/Ethnicity and Immigrant Generation*. Soc. Forces.

[31] StataCorp (2017). Stata Statistical Software: Release 15.

[32] Bruce M.A., Wilder T., Norris K.C., Beech B.M., Griffith D.M., Thorpe J.R.J. (2017). Perspective: Cardiovascular Disease among Young African American Males. Ethn. Dis..

[33] Mayne S.L., Hicken M.T., Merkin S.S., E Seeman T., Kershaw K.N., Do D.P., Hajat A., Roux A.V.D. (2018). Neighbourhood racial/ethnic residential segregation and cardiometabolic risk: The multiethnic study of atherosclerosis. J. Epidemiol. Community Health.

[34] Gooding H.C., Milliren C., Shay C.M., Richmond T.K., Field A.E., Gillman M.W. (2016). Achieving Cardiovascular Health in Young Adulthood—Which Adolescent Factors Matter?. J. Adolesc. Health.

[35] Griffith D.M. (2016). Biopsychosocial Approaches to Men’s Health Disparities Research and Policy. Behav. Med..

