# Supplementary material for: Racial Residential Segregation and Race Differences in Ideal Cardiovascular Health among Young Men
Source: Int J Environ Res Public Health. 2021 Jul 22;18(15):7755. doi: 10.3390/ijerph18157755 (PMC8345482; doi:10.3390/ijerph18157755)
Supplement: Supplementary file 1 [file ijerph-18-07755-s001.zip › ijerph-1275895-supplementary.pdf]

Table S1. Cardiovascular health: Ideal, intermediate, and poor thresholds of the Life's Simple 7 targets.

	**Data source: Add Health (Wave IV)**		
	Ideal	***Intermediate***	***Poor***
Diet	<4 sugary beverages/week	5-7 sugary beverages per week	8+ sugary beverages per week
Physical activity	5+ activities weekly	1-4 activities weekly	0 activities
Smoking	Never smoked regularly	Smoked in the past year	Current smoker
Body mass index	<25 kg/m^2	25 - 29.9 kg/m^2	30+ kg/m^2
Blood pressure	<120 SBP and <80 DBP (no medication use/no prior diagnosis)	SBP 120-139 or DBP 80-89 or treated to ideal level	> 140 SBP or > 90 DBP or treated to less than ideal
Total cholesterol	Bottom seven deciles (no medication use/no prior diagnosis)	8th and 9th deciles or treated to ideal	Top decile
Glucose	HbA1c<5.7% and fasting glucose <100 mg/dL or non-fasting glucose<200 mg/dL and no medication use/no prior diagnosis	5.7 - 6.4% HbA1c or 100-125 mg/dL fasting glucose	6.5+ HbA1c or 126+ fasting glucose or 200+ non-fasting glucose or diabetic medications
